# Crosstalk between ROS Homeostasis and Secondary Metabolism in *S. natalensis* ATCC 27448: Modulation of Pimaricin Production by Intracellular ROS

**DOI:** 10.1371/journal.pone.0027472

**Published:** 2011-11-17

**Authors:** Tiago Beites, Sílvia D. S. Pires, Catarina L. Santos, Hugo Osório, Pedro Moradas-Ferreira, Marta V. Mendes

**Affiliations:** 1 IBMC – Instituto de Biologia Molecular e Celular, Universidade do Porto, Porto, Portugal; 2 Departamento de Biologia, Faculdade de Ciências (FCUP), Universidade do Porto, Porto, Portugal; 3 ICBAS – Instituto de Ciências Biomédicas Abel Salazar, Universidade do Porto, Porto, Portugal; 4 IPATIMUP - Institute of Molecular Pathology and Immunology, University of Porto, Porto, Portugal; Universidad Nacional Autonoma de Mexico, Instituto de Biotecnologia, Mexico

## Abstract

*Streptomyces* secondary metabolism is strongly affected by oxygen availability. The increased culture aeration enhances pimaricin production in *S. natalensis*, however the excess of O_2_ consumption can lead to an intracellular ROS imbalance that is harmful to the cell. The adaptive physiological response of *S. natalensis* upon the addition of exogenous H_2_O_2_ suggested that the modulation of the intracellular ROS levels, through the activation of the H_2_O_2_ inducible catalase during the late exponential growth phase, can alter the production of pimaricin. With the construction of defective mutants on the H_2_O_2_ related enzymes SodF, AhpCD and KatA1, an effective and enduring modulation of intracellular ROS was achieved. Characterization of the knock-out strains revealed different behaviours regarding pimaricin production: whilst the superoxide dismutase defective mutant presented low levels of pimaricin production compared to the wild-type, the mutants defective on the H_2_O_2_-detoxifying enzymes displayed a pimaricin overproducer phenotype. Using physiological and molecular approaches we report a crosstalk between oxidative stress and secondary metabolism regulatory networks. Our results reveal that the redox-based regulation network triggered by an imbalance of the intracellular ROS homeostasis is also able to modulate the biosynthesis of pimaricin in *S. natalensis*.

## Introduction

*Streptomyces* are Gram-positive, filamentous, soil-dwelling bacteria well known for their ability to produce a wide variety of secondary metabolites [1]. The biosynthesis of secondary metabolites occurs in a growth-phase dependent manner and is controlled by environmental and physiological factors [2]. *Streptomyces* secondary metabolism is regulated by a complex network that integrates multiple factors and takes place at different levels: from the so-called pathway-specific regulatory genes to pleiotropic regulators which control both secondary metabolism and morphological differentiation.

Streptomycetes secondary metabolism is an aerobic process and thus affected by oxygen availability. However, high levels of molecular oxygen consumption can lead to the formation of reactive oxygen species - ROS (hydrogen peroxide, H_2_O_2_; superoxide radicals, O_2_^•−^ and hydroxyl radicals, HO^•^) that can damage cell components such as proteins, nucleic acids and lipids [3]. To counteract the toxic effects of ROS, microorganisms have developed an adaptive response that extends from the modulation of gene expression to changes in enzymatic and non-enzymatic activities. The molecular machinery activated by this adaptive response is able to sense, scavenge ROS and repair the molecular damage. Concomitantly, it has been suggested that ROS can play an important role as secondary messengers on cell signalling, based on reductive-oxidative mechanisms [4]–[6]. Among ROS, H_2_O_2_ is the best studied as signalling molecule.

The ability to maintain cellular redox balance is essential to all organisms and is mainly achieved by the conversion of the redox signals into regulatory outputs, usually at the transcription level, which allows adaptation to the altered environment. Several studies suggest that the consequences of the adaptive response to oxidative stress extend beyond the primary effect of defence into alterations in the secondary metabolism profile. Although stress-induced regulatory mechanisms have been globally studied in *Streptomyces*, at the present there is a lack of knowledge on the influence, at the molecular level, of oxidative stress on the production of secondary metabolites. The *S. coelicolor* JH11 (Δ*catR*) mutant strain that overproduces catalase (CatA), shows a reduced expression of the alkyl hydroperoxidase system (AhpCD) and produces lower amounts of actinorhodin [7]. Addition of a redox-cycling agent (phenazine methosulfate) to *S. clavuligerus* increases superoxide dismutase activity and also enhances clavulanic acid production by inducing the transcription of the pathway-specific regulator CcaR [8], [9]. The authors also report the same effect on the actinorhodin biosynthesis in *S. coelicolor* [9].

*Streptomyces natalensis* produces pimaricin, a 26-member tetraene macrolide antifungal antibiotic [10], widely used for the treatment of fungal keratitis and in the food industry to prevent mould contamination of non-sterile foods such as cheese, sausages, cured meat, among others. As a polyene, its antifungal activity lies in its interaction with membrane sterols, not causing membrane permeabilization as initially thought but inhibiting the sterol-dependent processes of membrane fusion and fission [11]. Pimaricin is synthesized by the action of a type I modular polyketide synthase (PKS) and its biosynthetic gene cluster has been previously sequenced and characterized [12]. The gene cluster contains 19 open reading frames including 5 multifunctional enzymes (PimS0-PimS4) that harbor 13 PKS modules [10], and 14 additional proteins involved in post-PKS modification of the polyketide skeleton (tailoring enzymes), export and regulation of gene expression [13]–[18]. Among these are two pathway-specific regulators, PimR and PimM. PimR is the archetype of a new class of regulators that combines an N-terminal domain corresponding to the SARP (*Streptomyes* antibiotic regulatory protein) family of transcriptional activators with a C-terminal homologous to guanylate cyclases and large ATP-binding regulators of the LuxR family (LAL)[13] . PimM combines an N-terminal PAS domain with a C-terminal helix–turn–helix (HTH) motif of the LuxR type [14], [19]. Both proteins seem to play an independent positive regulatory role in pimaricin biosynthesis by the transcriptional activation of different target genes. Additionally, a cholesterol oxidase encoding gene (*pimE*) is located in the centre of the cluster and although not classified as a pathway-specific regulator, it presents an important role, not yet fully understood, in the signaling transduction cascade leading to pimaricin biosynthesis [15].

Like in other streptomycetes, secondary metabolism in *S. natalensis* seems to be regulated in response to a variety of nutritional and environment signals in a growth-phase dependent manner [20]. In this study we present evidence for a functional molecular crosstalk between ROS homeostasis and secondary metabolism in *S. natalensis*. Using a combined approach of physiological and molecular characterization we present evidence that intracellular H_2_O_2_ levels are important to elicit pimaricin biosynthesis, particularly during late exponential phase. Modulation of intracellular H_2_O_2_ levels, either by prompting an adaptive response of *S. natalensis* to H_2_O_2_-induced oxidative stress or by the construction of knock-out mutants on the main H_2_O_2_-related enzymes, altered the pimaricin production profile.

## Results

### *S. natalensis* presents a catalase activity profile dependent on the growth-phase

**Figure 1:**
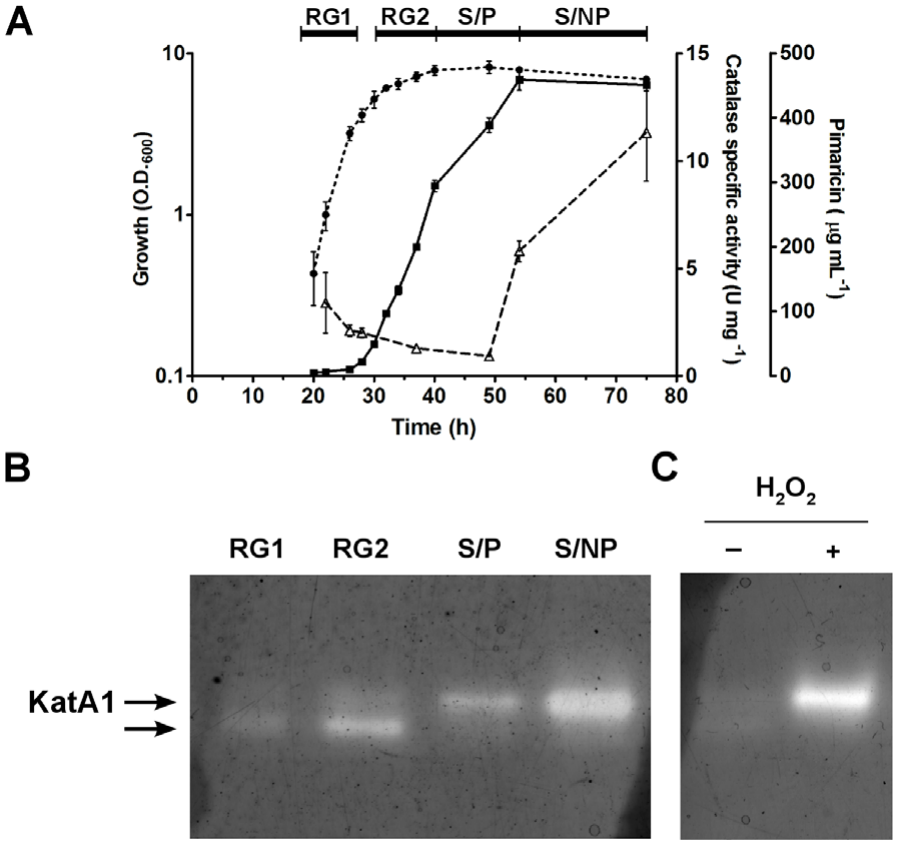
Pimaricin production and antioxidant growth-dependent profile of *S. natalensis*. (A) Growth (··•··), catalase activity (–Δ–) and pimaricin production (−▪−) of *S. natalensis* ATCC 27448 in YEME medium. Growth phases are indicated by solid lines at the top of the graph. Vertical bars indicate standard deviation of the mean values. Data are the average of triplicates from three independent experiments. (B)Native PAGE of cell extracts (30 µg protein per lane) from *S. natalensis* stained for catalase activity. *S. natalensis* was grown in YEME medium and samples were collected during the defined four growth phases (see Material and Methods): RG1, RG2, S/P and S/NP. Arrows show the two detectable catalase activity bands. (C) Native PAGE stained for catalase activity of cell extracts from *S. natalensis* ATCC 27448 cultures collected during RG1 phase after 1 mM H_2_O_2_ insult (+).

In YEME liquid medium *S. natalensis* ATCC 27448 presents a typical growth curve, pimaricin is first detected during the late exponential phase and its production occurs until mid-stationary phase (Fig. 1A). For experimental purposes and in agreement to what was previously described for *S. coelicolor* [21], [22], the *S. natalensis* growth curve was divided into four growth stages: an early exponential phase characterized by a rapid growth (RG1); after a brief transition phase linked with the “metabolic switch” [23], there is a second rapid growth phase (RG2) with a lower growing rate that overlaps with the late exponential phase. Afterwards the cultures enter into the stationary phase (S). We have divided the stationary phase into an early- to mid-stationary phase when pimaricin biosynthesis occurs (S/P), and a late stationary phase, when pimaricin is no longer being synthesized by *S. natalensis* (S/NP) (Fig. 1A).

Regarding ROS homeostasis, catalases are key enzymes that have the ability to scavenge H_2_O_2_. The catalase activity profile of *S. natalensis* was monitored along the growth curve in YEME liquid medium (Fig. 1A). Total catalase activity gradually decreased during the exponential phase and at early stationary phase presented low levels (<2 U mg^−1^). These low levels of catalase activity were maintained until mid-stationary phase and then a continuous increase was observed until the late stationary phase. Like *S. coelicolor* [24], [25] and *S. avermitilis* (Fig. S1), *S. natalensis* presents a growth-dependent behaviour of catalase activity. However, whereas *S. coelicolor* and *S. avermitilis* present a constant increase in catalase activity from the early exponential to the late stationary phases, *S. natalensis* profile showed low levels of catalase activity during RG2 and S/P phases, the pimaricin production phases. Furthermore, when compared to other *Streptomyces* strains, in particular *S. coelicolor* and *S. avermitilis*, the overall levels of total catalase activity in *S. natalensis* are considerably lower (Fig. S1).

Cell free protein extracts were also analysed by catalase activity staining after native PAGE (Fig. 1B). Two activity bands were detectable suggesting the expression of at least two catalase enzymes in *S. natalensis* under the conditions tested (Fig. 1B). A lower molecular weight band whose intensity increased during exponential phase up to RG2 phase and disappeared as the culture entered into the stationary phase. During stationary phase, a new activity band was induced contributing to the increase in total catalase activity quantified spectrophotometrically. When gels were stained for peroxidase activity, no activity was detected under the conditions tested (data not shown). To further confirm the absence of a bifunctional catalase peroxidase encoding gene, a common H_2_O_2_-dismutating enzyme in this bacterial group, a Southern and an immunoblot analysis were performed on genomic DNA (an internal probe from *S. coelicolor* catalase-peroxidase encoding gene was used) and on cytoplasmatic and extracytoplasmatic protein extracts from *S. natalensis* (using a primary antibody raised against a catalase-peroxidase purified from *S. reticuli* [26]) respectively. In both cases no positive signal was detected (data not shown).

### Concerted regulation of katA1 and ahpC in response to H_2_O_2_

**Figure 2:**
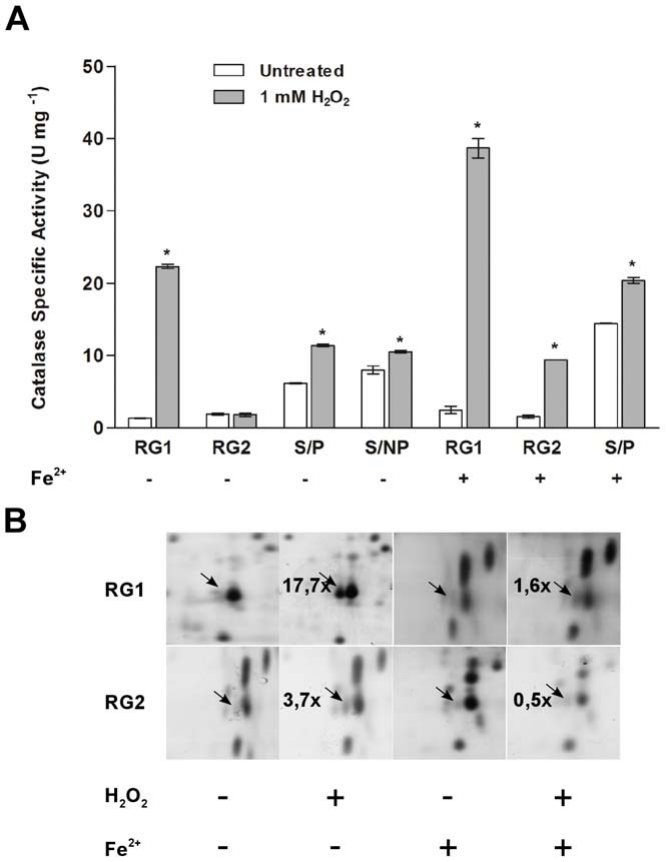
*S. natalensis* response to H_2_O_2_ induced stress. (A) Induction of catalase activity by addition of H_2_O_2_ at different growth stages in control and iron supplemented cultures. Protein extracts were prepared from *S. natalensis* cultures collected 1 hour after 1 mM H_2_O_2_ (final concentration) treatment or an equal volume of water as the untreated control. Induction of catalase activity by H_2_O_2_ treatment was assessed at the four defined growth stages (see Material and Methods): RG1, RG2, S/P and S/NP. Results (average of triplicates and standard deviation) are representative of three independent experiments. The differences between treated and untreated samples at each time point were assessed by independent t-test. *, statistically significant (P<0.01). (B) Induction of AhpC expression by H_2_O_2_ induced stress. Protein extracts were analysed by comparative 2D gel electrophoresis to assess the expression profile of the *S. natalensis* proteome under induced stress conditions. The protein profile of the identified AhpC spot (indicated by an arrow) is enlarged. Fold variations of the AhpC spot between H_2_O_2_-treated and untreated samples are indicated in the top left of the H_2_O_2_-treated panels. Fold variations reflect the 2D-gel analysis of three independent experiments.

To study the response of *S. natalensis* ATCC 27448 to exogenous H_2_O_2_, total specific catalase activity was measured in cultures challenged with H_2_O_2_. Three different H_2_O_2_ concentrations were tested, 0.1 mM, 1 mM and 10 mM. Addition of 10 mM H_2_O_2_ at RG1 phase severely impaired growth of *S. natalensis*, and catalase activity induction with 1 mM H_2_O_2_ was 1.7-fold higher than with 0.1 mM (data not shown). Therefore, studies of catalase activity inducibility were carried out adding 1 mM H_2_O_2_ to the culture broth at RG1, RG2, S/P and S/NP phases. Specific catalase activity was measured in protein extracts collected 1 h after H_2_O_2_ insult (Fig. 2A). Addition of H_2_O_2_ prompted an increase in total catalase activity except in the RG2 phase where no induction of catalase activity was observed. Additionally, analysis of the protein extracts from RG1 phase by native PAGE revealed that the addition of H_2_O_2_ induces the catalase that is active during the stationary phase (Fig. 1C).

To further analyze the changes triggered by H_2_O_2_ at the proteome level two dimensional (2D) gel electrophoresis was carried out to compare the proteome of H_2_O_2_-treated and non-treated cultures. Protein expression differences of the 2D gels were analyzed using appropriate software tools (PDQuest, Biorad). The analysis revealed 43, 81, 121 and 39 differences in the RG1, RG2, S/P and S/NP phases, respectively. Among those changes it was detected, in H_2_O_2_-treated cultures, an 17.7- (during RG1 phase) and an 3.7-fold (in RG2 phase) induction of the alkyl hydroperoxide reductase (AhpC) protein, a component of the alkyl hydroperoxidase reductase system which exhibits activity against H_2_O_2_, organic peroxides and peroxynitrite (Fig. 2B). These results suggest that the H_2_O_2_ added at RG2 phase was detoxified through the action of the alkyl hydroperoxidase reductase system.

**Figure 3:**
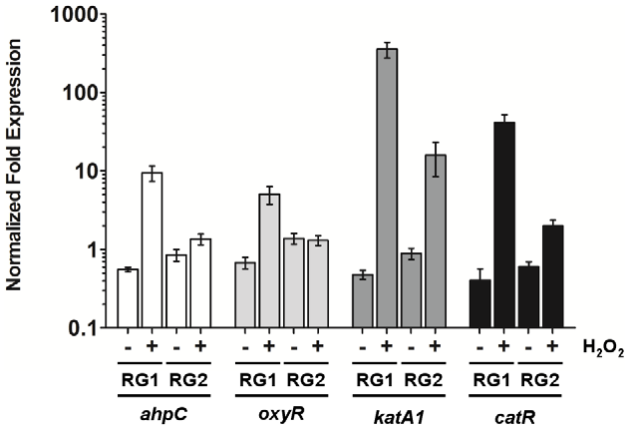
Transcriptional analysis of *ahpC*, *oxyR*, *katA1* and *catR.* Transcription profiles of the monofuncional catalase (*katA1*) and alkyl hydroperoxide reductase (*ahpC*) encoding genes and their transcriptional regulators, *catR* and *oxyR*, in *S. natalensis* ATCC 27448 upon a H_2_O_2_ insult. The transcription of ahpC, *oxyR*, *katA1* and *catR* was evaluated by RT-qPCR from *S. natalensis* grown in YEME medium and samples collected 30 min after 1 mM H_2_O_2_ treatment (+) or an equal volume of water as the untreated control (-).H_2_O_2_ treatments were applied independently during the early exponential phase (RG1) and late exponential phase (RG2). The Mean Normalized Fold Expression (±standard errors) of the target genes was calculated relative to the transcription of the reference genes (16 S rDNA and *lysA*) and the reaction internal normalization was performed using the sample from cells collected immediately before H_2_O_2_ addition to the culture broth (not shown). Results (average of triplicates and standard deviation) are representative of three independent experiments.

In *S. coelicolor*, the induction of the catalase by H_2_O_2_ is regulated in an iron-dependent manner by the fur-like protein CatR [7]. To assess the sensitivity of catalase induction to H_2_O_2_ in the presence of an excess of iron, total catalase activity was measured in iron-supplemented (20 µM FeSO_4_) cultures with and without the addition of H_2_O_2_ (Fig. 2A). Under these conditions, during RG1 and S/P phases the same behaviour was observed regarding catalase response to H_2_O_2_. However, induction of catalase activity was also observed at the RG2 phase whereas the protein levels of AhpC did not change significantly (Fig. 2B). These results suggest an iron dependent crosstalk between the regulation of these two main H_2_O_2_ scavenging enzymes. RT-qPCR analysis was performed to confirm this concerted regulation between the catalase and the alkyl hydroperoxidase reductase system (Fig. 3). The results showed that the increase in total catalase activity during the exponential phase was due to an increase of the transcript levels of *katA1*. The transcripts of the H_2_O_2_-sensing regulator *catR* were also increased, suggesting the presence of a similar mechanism to that of *S. coelicolor* [7]. The *ahpC* and *oxyR* (the H_2_O_2_-sensing transcriptional regulator of the AhpCD system; see below) transcriptional profiles in iron-supplemented cultures (Fig. 3), are reflected in the AhpC protein expression profile (Fig. 2B) i.e. in the presence of iron, during RG2 phase, H_2_O_2_ was detoxified mainly by the action of the catalase.

### The adaptive response triggered by H_2_O_2_ modulates pimaricin biosynthesis

**Figure 4:**
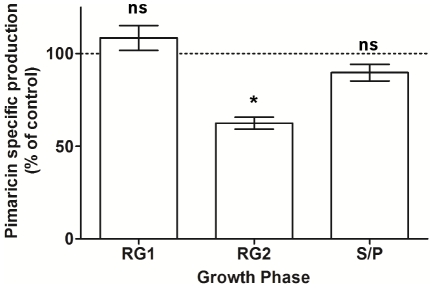
Pimaricin specific production in H_2_O_2_ growth dependent induced cultures. *S. natalensis* ATCC 27448 was grown in iron-supplemented YEME medium, and pimaricin specific yield (per mg of total protein) measured at 72 h. 1 mM (final concentration) H_2_O_2_ was added to the culture broth either at the RG1, RG2 or S/P phase. Data are the means from three independent experiments. To assess the presence of significant differences between the tested growth phases, a one-way ANOVA was performed followed by post-hoc test (Tukey test; GraphPad Prism) in which each condition was compared to the control situation (pimaricin production without H_2_O_2_ addition; 100%). *, statistically significant (P<0.01); ns, not statistically significant.

The adaptive response prompted by the addition of H_2_O_2_ in iron-supplemented cultures reflected in an increase in the total catalase activity, particularly during exponential phase. In *E. coli*, catalase is described as a more efficient enzyme at high levels of H_2_O_2_, whilst AhpC being a more efficient scavenger of trace H_2_O_2_ generated endogenously [27]. Induction of total catalase activity in iron-supplemented cultures reflected in a transient and reversible decrease in H_2_O_2_ intracellular levels, particularly during RG2 phase when a decrease of 25.6% in H_2_O_2_ intracellular concentration, 1 h after the H_2_O_2_ insult, was observed (data not shown). To determine whether the pimaricin production in *S. natalensis* is affected by the temporary redox imbalance created by the adaptive response to H_2_O_2_, pimaricin yields at 72 h were measured in the culture broths of H_2_O_2_-induced iron supplemented cultures (Fig. 4). When the H_2_O_2_ stimulus was introduced during RG1 phase no significant alteration in pimaricin yield was observed. However, when H_2_O_2_ stimulus was introduced during late exponential phase (RG2) the pimaricin yield was reduced to 62.5% of the production in the control culture (without the H_2_O_2_ stimulus). Although less pronounced, a decrease in pimaricin production (ca 10%) was also observed in cultures with H_2_O_2_-stress induced at S/P phase.

### Cloning of genes related with H_2_O_2_ from *S. natalensis*

**Figure 5:**
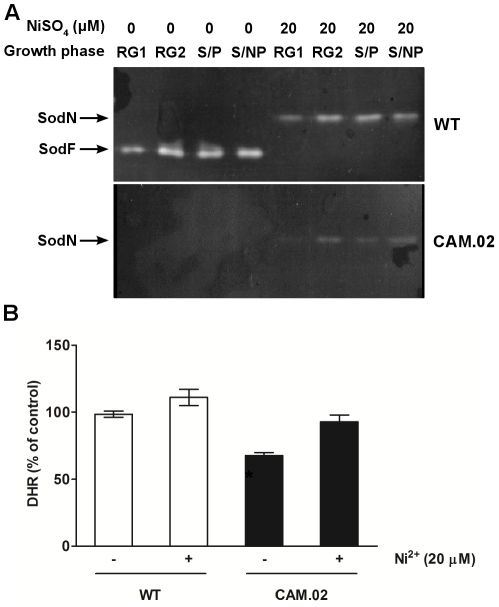
Lack of SOD activity in strain CAM.02 decreases intracellular H_2_O_2_ levels. (A) Native PAGE of cell extracts (30 µg protein per lane) from *S. natalensis* ATCC 27448 (upper panel) and *S. natalensis* CAM.02 (lower panel) stained for SOD activity. *S. natalensis* strains were grown in YEME medium and cells collected on the four previously defined growth stages. NiSO_4_ 20 µM (final concentration) was added to the YEME medium for the induction of *sodN* expression. (B) Intracellular H_2_O_2_ levels in *S natalensis* ATCC 27448 (WT) and SodF defective mutant (CAM.02) at RG2 growth phase. Values are means from two independent experiments. To assess the presence of significant differences between the tested condition, a one-way ANOVA was performed followed by post-hoc test (Tukey test; GraphPad Prism) in which each condition was compared to the control situation (wild-type strain intracellular H_2_O_2_ levels in Ni non-supplemented cultures; 100%). *, statistically significant (P<0.01).

The results from the native PAGE and 2D analyses showed the existence in the *S. natalensis* ATCC 27448 genome of genes coding for at least one H_2_O_2_ inducible monofunctional catalase (Fig. 1B), a Fe and Ni-containing SOD (Fig. 5A) and an alkyl hydroperoxide reductase (Fig. 2B). To clone those encoding genes we screened a *S. natalensis* ATCC 27448 cosmid library [10] with probes KA1, internal to *katA1*, AHP internal to *ahpC* and SF internal to *sodF* (see Materials and Methods).

A 2.4 kb *Nco* I fragment from cosmid A1, was found to hybridize with probe KA1. The fragment was cloned into *Nco* I-digested pGEM^®^-T Easy vector and sequenced. The fragment was 2430 bp and *in silico* analysis of the fragment showed the presence of two complete open reading frames (ORFs), *katA1* (1464 bp) and *catR* (423 bp), divergently transcribed and separated by 161 bp (Fig. S2A). Both genes presented an overall codon usage pattern in good agreement with that of typical *Streptomyces* genes. The product of *katA1* (487 amino acids with a deduced molecular mass of 54 kDa) showed high sequence identities with other *Streptomyces* monofunctional catalases particularly with the whole protein of SAV_3052 (87% identity), a putative catalase from *S. avermitilis* MA-4680. Like most monofunctional catalases, two conserved domains are identified in KatA1: in the N-terminal region of the protein it is found the catalase active site motif (amino acids 47 to 63) and in the C-terminal region the heme-ligand motif (amino acids 337 to 345). Upstream and divergently transcribed from *katA1*, *catR* was identified. CatR protein is 140 amino acids long and showed high sequence identity with other *Streptomyces* PerR like members of the Fur family of proteins (78% to 83% identity), in particular with hydrogen peroxide sensitive repressors. It contains the four cysteine residues conserved in PerR like proteins (C^92^-X_2_-C^95^ and C^132^-X_2_-C^135^) involved in structural Zn^2+^ binding as well as the two histidine residues (H^32^ and H^87^) involved in Fe^2+^ (or Mn^2+^) coordination [28], [29].

A 1.8 kb *Apa* I fragment from cosmid SF6 was found to hybridize with probe SF internal to *sodF*. The fragment was cloned into *Apa* I-digested pGEM^®^-T Easy vector and sequenced. The fragment was 1775 bp and *in silico* analysis of the fragment showed the presence of one complete ORF, *sodF* (642 bp) (Fig. S3A). Comparison of the protein encoded by *sodF* (213 aa) with the non-redundant protein sequence database (NCBInr) revealed high sequence identity (91 to 95% identity over the whole protein sequence) with FeZn superoxide dismutase proteins from *Streptomyces*. Analysis of the deduced amino acid sequence of SodF revealed the presence of the four conserved residues involved in binding to the metal cofactor (H^28^, H^76^, D^165^ and H^169^) [30], two of them included in the SOD conserved motif [D^165^-A-W-E-H-A-F-Y^172^] [31]. Furthermore, the upstream genomic region of the *sodF* gene revealed the presence of the Ni-responsive regulatory motif (TTGCAN_7_TGCAA) suggested to be involved in the nickel dependent transcriptional repression of *sodF* [32].

A 3.75 kb *Bam* HI fragment from cosmid X10 was found to hybridize with probe AHP internal to *ahpC*. The fragment was cloned into *Bam* HI-digested pUC19 vector and sequenced. The nucleotide sequence analysis of the fragment revealed three complete ORFs: *ORF1* (627 bp), *oxyR* (960 bp), *ahpC* (555 bp) and *ahpD* (534 bp) (Fig. S4A). The deduced protein sequences of *oxyR*, *ahpC* and *ahpD* showed high sequence identities with counterparts from *Streptomyces*, in particular a hydrogen peroxide sensing regulator of the LysR-family, an alkyl hydroperoxide reductase and an alkylhydroperoxidase respectively. The three conserved catalytic cysteine residues described to be involved in the peroxidatic activity of *Mycobacterium tuberculosis* AhpC [33] were identified in *S. natalensis* AhpC (C^51^, C^164^ and C^166^). Additionally, the conserved motif CXXC of electron transport proteins was identified in *S. natalensis* AhpD (C^131^ and C^134^). *ORF1* codes for a putative protein of unknown function that shows high identity with protein SGR3203 from *S. griseus*.

Knock-out mutants on the identified anti-oxidant encoding genes were constructed by replacing the target gene with an apramycin resistance cassette using the PCR targeting method (see Material and Methods) [34]. Three mutant strains were isolated: CAM.02 (Δ*sodF*::*aac(3)IV-oriT*), CAM.04 (Δ*ahpCD*::*aac(3)IV-oriT*) and CAM.05 (Δ*katA1*::*aac(3)IV-oriT*) (Table 1) and their identity was confirmed by Southern blot and PCR analyses (Supplementary material; Fig. S2, S3, S4). In the case of CAM.05 (Δ*katA1*) mutant, to further confirm the identity of KatA1 as being inducible by H_2_O_2_, CAM.05 strain was challenged with a H_2_O_2_ insult during RG1 phase. The mutant strain was unable to induce catalase activity confirming that the catalase encoded by *katA1* is inducible by H_2_O_2_ (data not shown). Antifungal activity and the identity of pimaricin produced by the knock-out mutants were confirmed by bioassay and ultra performance liquid chromatography (UPLC), respectively (data not shown).

**Table 1:** Strains and plasmids used in this study.

Strain or plasmid	Description	Reference
**Strain**		
*S. natalensis*		
ATCC 27448	Wild-type strain	[62]
CAM.02	*sodF* loci replaced by the *oriT-aac(3)IV* cassette; Δ*sodF*::*aac(3)IV-oriT*	This study
CAM.04	*ahpCD* loci replaced by the *oriT-aac(3)IV* cassette; Δ*ahpCD*::*aac(3)IV-oriT*	This study
CAM.05	*katA1* loci replaced by the *oriT-aac(3)IV* cassette; Δ*katA1*::*aac(3)IV-oriT*	This study
*E. coli*		
DH5α	General cloning strain	[63]
ET12567	Non-methylating strain used for conjugation with *Streptomyces*	[64]
BW25113	Strain used for PCR-targeted mutagenesis	[65]
BT340	DH5a [pCP20]	[66]
**Cosmids & Plasmids**		
pGEM-T Easy	General cloning vector	Promega
pSET152neo	pSET152 derivative, *neo*	[17]
pIJ773	*oriT aac(3)IV*	[34]
pUZ8002	Mobilization plasmid; *neo*	[67]
A1	Cosmid from genomic library of *S. natalensis* ATCC 27448; *katA1 catR*	This study
SF6	Cosmid from genomic library of *S. natalensis* ATCC 27448; *sodF*	This study
X10	Cosmid from genomic library of *S. natalensis* ATCC 27448; *oxyR ahpCD*	This study

All three mutants grew normally in liquid and solid media. However, the CAM.05 mutant was unable to form aerial mycelia and to sporulate in solid media. This phenotypic trait hampered strain manipulation, in particular mutant complementation and pimaricin production assays. DNA delivery methodologies such as transformation or intergeneric conjugation using mycelium have been unfruitful for *S. natalensis* [35] and additionally, pimaricin production yields are significant lower when vegetative mycelium is used as inoculum [36]. This morphological phenotype is different from that reported for the *S. coelicolor* mutant strain YD9147, a *catA* defective mutant [37], that sporulated normally on R2YE medium.

### Loss of SOD activity decreases pimaricin production

Superoxide dismutase (SOD) enzymes catalyze the dismutation of the superoxide anion radical into H_2_O_2_ and O_2_. Two types of SODs have been characterized in *Streptomyces*, a nickel containing SOD (NiSOD) and both iron and zinc containing SOD (FeZnSOD) [38], [39]. Native PAGE analysis of *S. natalensis* crude extracts (Fig. 5A) showed a band with SOD activity corresponding to a FeZnSOD expressed constitutively throughout the growth curve. It has been reported a nickel dependent regulatory system that controls the expression of either the NiSOD or the FeZnSOD in *S. coelicolor* and *S. griseus*, i.e., in the presence of nickel ions NiSOD is preferentially expressed whereas FeZnSOD expression is down-regulated [39], [40]. The presence of the Ni-responsive regulatory motif upstream from *sodF* suggests a similar regulation mechanism in *S. natalensis*. Indeed, in NiSO_4_ (20 µM) supplemented cultures, a second band displaying SOD activity can be observed in *S. natalensis* (Fig. 5A) suggesting the presence of a NiSOD. Moreover, the transcription of *sodF* was repressed in Ni-supplemented cultures as assessed by RT-qPCR (data not shown). When protein extracts from the *sodF* defective mutant (CAM.02) were analyzed by native PAGE, no FeZnSOD band was detected but it was still observed the band corresponding to the NiSOD in Ni-supplemented cultures (Fig. 5A).

SOD enzymes are key enzymes involved in intracellular ROS homeostasis. Deleting the FeZnSOD encoding gene created an imbalance in the intracellular ROS concentration of CAM.02 strain, throughout the growth curve. In particular, intracellular H_2_O_2_ levels at RG2 phase (when pimaricin biosynthesis begins) decreased 33% in CAM.02 mutant when compared to the wild-type. Ni-supplementation of the CAM.02 culture broth restored H_2_O_2_ to wild-type levels (Fig. 5B).

Quantification of pimaricin at 72 h in culture broths of CAM.02 strain grown in YEME medium, showed that the pimaricin specific production of the mutant strain was 8.2% of the wild-type. Interestingly, the pimaricin specific production was increased in CAM.02 mutant grown in Ni-supplemented medium (34.3% of the wild-type). Complementation of the CAM.02 with *sodF* under its own promoter restored pimaricin production to wild-type levels (Table 2).

**Table 2:** Pimaricin production of *S. natalensis* mutant strains.

*S. natalensis* strain (genotype)	Conditions	Pimaricin production(% of WT)^a^
CAM.02 (Δ*sodF:: oriT-aac(3)IV*)	-	8.2±1.45
	20 µM NiSO_4_	34.3±0.99
	pSET-*sodF*	86.0±0.22^b^
CAM.04 (Δ*ahpCD:: oriT-aac(3)IV*)	-	129.9±4.66
	20 µM Fe^2+^	156.3±5.60
	10 mM H_2_O_2_ @ RG2	169.4±7.82
	20 µM Fe^2+^ + 10 mM H_2_O_2_ @ RG2	174.1±0.23
	pSET-*ahpCD*	101.2±0.76^b^
CAM.05 (Δ*katA1:: oriT-aac(3)IV*)	-	156±4.05^c^
	1 mM H_2_O_2_ @ RG2	203.8±9.41^c^

a Values are means of triplicates from three independent experiments; 100% represents the pimaricin production of *S. natalensis* ATCC 27448 at 72 h in YEME medium.

b 100% represents pimaricin production of *S. natalensis* harbouring pSET152neo at 72 h in YEME medium.

c Pimaricin production of *S. natalensis* CAM.05 strain was assessed at 72 h in YEME medium inoculated with vegetative mycelium. 100% represents the pimaricin production of *S. natalensis* ATCC 27448 at 72 h in YEME medium inoculated with vegetative mycelium.

### Mutants defective in either H_2_O_2_ detoxification enzymes show a pimaricin overproducer profile

To counteract the increase in intracellular H_2_O_2_ levels, microorganisms have enzymes that present H_2_O_2_-scavenging activity such as catalases and the alkyl hydroperoxide reductase system. The *S. natalensis* mutants defective on such H_2_O_2_-detoxifying enzymes, CAM.04 (Δ*ahpCD*) and CAM.05 (Δ*katA1*) showed different phenotypic traits but both behaved as pimaricin overproducers, reaching 130% and 156% of the pimaricin produced by the wild-type strain, respectively (Table 2). Also, it is worth noting that the pimaricin overproducer behaviour of the mutant strains, CAM.04 and CAM.05, was enhanced by the addition of H_2_O_2_ to the culture broths during RG2 phase (Table 2). Finally, complementation of the CAM.04 with *ahpCD* under its own promoter restored pimaricin production to the wild-type levels (Table 2).

**Figure 6:**
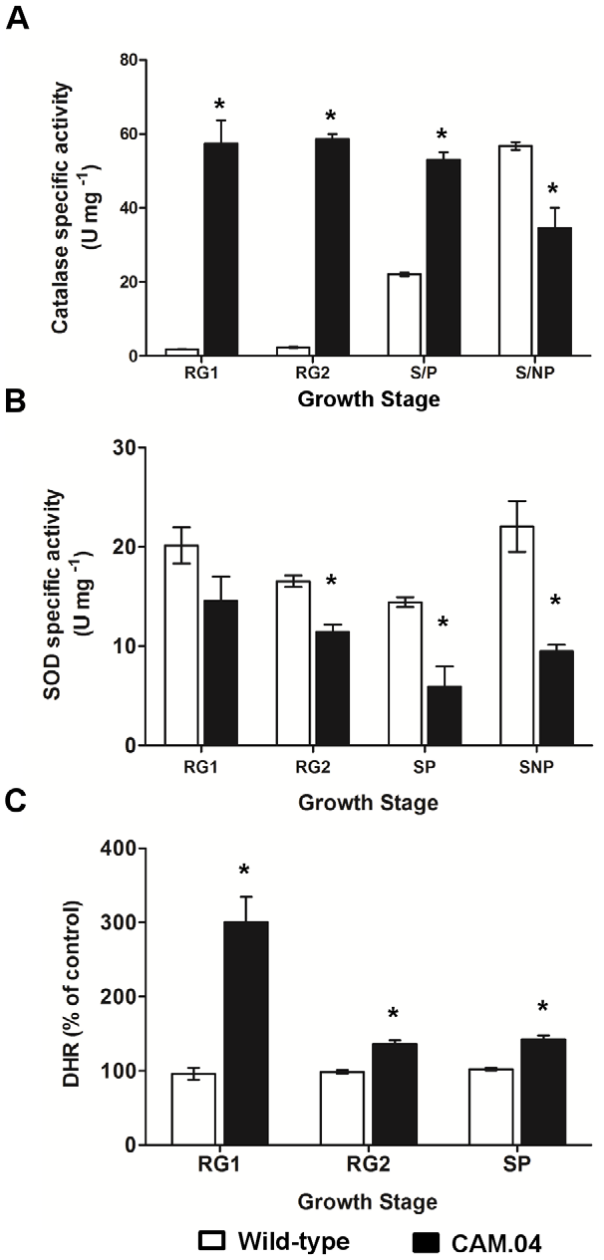
Characterization of *S. natalensis* CAM.04 strain. (A) Total catalase specific activity, (B) SOD specific activity and (C) intracellular H_2_O_2_ levels of *S. natalensis* wild-type (white bars) and *S. natalensis* CAM.04 (black bars) at the four defined growth stages: RG1, RG2, S/P and S/NP. Results (average of triplicates and standard deviation) are representative of three independent experiments. The presence of statistically significant differences between wild-type (WT) and CAM.04 samples was determined by t-test. *, statistically significant (P<0.01).

The intracellular H_2_O_2_ levels of the *S. natalensis* CAM.04 (Δ*ahpCD*) strain were higher than those of the wild-type strain, particularly during RG1 phase with a 3-fold increase (Fig 6C). During RG2 and S/P growth phases the H_2_O_2_ levels were 30% to 40% above the wild-type strain levels (Fig. 6C). Concomitantly with the high H_2_O_2_ intracellular levels, the total catalase specific activity, namely during the exponential growth phases (RG1 and RG2), had a significant boost, reaching a 30-fold increase in RG1 phase when compared to the wild-type (Fig. 6A). Native PAGE analysis showed that the increase in catalase activity is due to the increase of the levels of the catalase inducible by H_2_O_2_ (data not shown). Regarding total specific SOD activity, CAM.04 showed a similar profile to the wild-type, although with lower activities (Fig. 6B).

The high levels of intracellular H_2_O_2_ present a vulnerability to the CAM.04 strain because of the H_2_O_2_ reduction to the highly toxic HO^•^ by the metal-catalysed Fenton reaction. To determine the outreach of such toxicity regarding pimaricin biosynthesis, CAM.04 strain was grown in iron-supplemented medium and pimaricin specific production was assessed with and without the addition of H_2_O_2_ to the culture broth (Table 2). In both situations the overproducer phenotype of the CAM.04 strain persisted (174% and 156% of the pimaricin produced by the wild-type, respectively).

## Discussion

Optimization of the strain ideal aeration conditions in submerged cultures is an essential step of industrial fermentation processes as microbial secondary metabolism is strongly affected by oxygen availability. Previous studies have shown that pimaricin production is highly dependent on dissolved oxygen levels, namely an increase in culture aeration lead to higher yields of pimaricin [36]. However, an increased consumption of O_2_ can generate ROS that would trigger an adaptive response in order to sense and scavenge ROS and repair the damage in cell components. In this work we addressed the role of ROS homeostasis, in particular intracellular H_2_O_2_ levels, and of the adaptive response triggered by H_2_O_2_, on the production of pimaricin by *S. natalensis* ATCC 27448.

Catalases play a key role in ROS homeostasis due to their ability to perform the dismutation of H_2_O_2_ into water and molecular oxygen. Analysis of catalase activity in crude extracts of *S. natalensis* showed that, under the conditions tested, two different catalases contribute to the specific total catalase activity profile presented by *S. natalensis*. The expression of both catalases is growth-phase dependent (Fig. 1B): while one catalase was mainly active during the exponential growth phases (RG1 and RG2) the second catalase was present during the stationary phase. Characterization of the response of *S. natalensis* wild-type and CAM.05 (Δ*katA1*) strains to exogenous H_2_O_2_ allowed us to confirm that the stationary phase specific catalase from *S. natalensis* (KatA1) is inducible by H_2_O_2_. However, its behaviour regarding the response to H_2_O_2_ was considerably different from that of *S. coelicolor* [24], [41]. During RG2 phase no induction of catalase activity was observed upon the addition of H_2_O_2_. Instead, an increase in the expression of the alkyl hydroperoxide reductase protein (AhpC) suggested a detoxification of H_2_O_2_ by the alkyl hydroperoxide system (AhpCD). Conversely, in iron supplemented cultures, an induction of total catalase activity is achieved during RG2 phase, whereas no induction of the AhpC expression is observed. Thus, in the presence of an excess of iron, H_2_O_2_ was being detoxified mainly by the action of the monofunctional catalase, KatA1. In *S. coelicolor,* the two main H_2_O_2_ scavenging enzymes (CatA and AhpCD) are regulated independently by two proteins able to sense H_2_O_2_, CatR and OxyR [42], respectively. During the early exponential phase (before the metabolic switch) and stationary phase, *S. natalensis* counteracts the addition of H_2_O_2_ by inducing catalase activity. However, during the late exponential phase (RG2), in order to maintain H_2_O_2_ homeostasis, *S. natalensis* activates the AhpCD system upon a H_2_O_2_ challenge instead of catalase, which has been described as a more effective enzyme at high concentrations of H_2_O_2_ [27].

Assuming similar tertiary structures between the H_2_O_2_-sensing regulators from *S. natalensis* (CatR_SNA_) and *S. coelicolor* (CatR_SCO_) and PerR from *Bacillus subtilis* , and taking into consideration the postulated models for CatR_SCO_ and PerR DNA binding mechanisms [7], [28], the behaviour of CatR_SNA_ in iron-supplemented experiments led us to hypothesise about its mechanism. We suggest that the mechanism of sensing H_2_O_2_ and DNA binding activity of CatR_SNA_ is not through disulfide bond formation between the C-terminal cysteine residues, as proposed for *S. coelicolor* [7], but in a metal-dependent manner through the metal-catalyzed oxidation (MCO) of the histidine residues (H^32^ or H^87^), responsible for iron coordination as postulated for PerR in *B. subtilis* [28]. The higher availability of Fe^2+^ in iron-supplemented cultures increases the interaction of CatR_SNA_ with Fe^2+^, enhancing CatR_SNA_ susceptibility to MCO by H_2_O_2_ thus increasing *katA1* transcription. However, this needs to be further investigated experimentally to fully elucidate the CatR_SNA_ H_2_O_2_ sensing and DNA binding mechanism.

The induction of KatA1 activity during RG2 phase by addition of H_2_O_2_ in iron-supplemented cultures lowered the intracellular H_2_O_2_ levels. However the effects of catalase induction went beyond the adaptive response towards H_2_O_2_ and reflected in a decrease in the specific production of pimaricin by *S. natalensis*. Although it was observed a growth phase dependent response, regarding pimaricin biosynthesis, upon the addition of H_2_O_2_ in iron-supplemented cultures, the harmful effect of hydroxyl radicals generated by the Fenton reaction may also contribute to the decrease in the pimaricin specific production during RG2 phase. Nevertheless, these results suggest that the intracellular levels of ROS are able to modulate the biosynthesis of pimaricin. Previous studies had suggested that ROS, in particular H_2_O_2_, play an important role as secondary messengers on cell signalling based on reductive-oxidative mechanisms [4], [43]. We hypothesize that in iron-supplemented cultures, the “forced” activation of a monofunctional catalase during RG2 phase, detoxified H_2_O_2_ to levels below those of the wild-type strain, altering the secondary metabolism regulatory network at the pleiotropic or at the pathway-specific level. Interestingly, the pimaricin biosynthetic gene cluster in *S. natalensis* comprises the positive regulator PimM which has an N-terminal PAS domain [14]. PAS sensor domains are able to sense a wide range of signals including cellular redox status [44]. This feature turns PimM into a possible (in)direct target of a redox-dependent regulation.

To test the possibility of a regulation of secondary metabolism by intracellular H_2_O_2_, a functional approach was pursued with the construction of knock-out mutants in genes coding for hydrogen peroxide related enzymes. The construction of the mutant strains defective in anti-oxidative enzymes (SodF, KatA1 or AhpCD), allowed an effective modulation in H_2_O_2_ intracellular concentration in contrast to the punctual imbalance created by the addition of H_2_O_2_ to the culture broth that elicits an adaptive response to restore normal cell redox status.

An important source of intracellular H_2_O_2_ is the dismutation of superoxide anion by SOD. In fact, the SodF defective mutant, *S. natalensis* CAM.02, showed lower levels of intracellular H_2_O_2_ along the growth phase, in particular during RG2 phase with a 30% decrease (Fig. 5B). Simultaneously, a drastic decrease in the production of pimaricin was also observed when compared to the wild-type. In contrast, supplementation of the culture broth with Ni^2+^ ions not only increased the expression of the Ni-containing SOD but also, presumably as a result of the NiSOD action, increased the intracellular H_2_O_2_ levels. These conditions increased the pimaricin specific production showing a possible correlation between H_2_O_2_ intracellular levels and pimaricin production in *S. natalensis*. However it is expected an increase in the intracellular levels of the superoxide anion in the CAM.02 mutant. As a consequence, part of the negative effect observed in the pimaricin production could be related to the toxic effect of the superoxide anion particularly in the proteins containing iron-sulphur clusters. Recently, heterologous expression of a FeZnSOD and NiSOD from *S. peucetius* in *S. clavuligerous* and *S. lividans* increased the production of clavulanic acid and actinorhodin respectively [45]. The authors ascribe a positive effect of SODs in secondary metabolite production. The results obtained in *S. natalensis* lead us to go further on and suggest that the positive effect observed was due to an increase in the intracellular H_2_O_2_ levels.

Increased levels of intracellular H_2_O_2_ were observed, particularly during the exponential phase, in the H_2_O_2_ scavenging defective mutant, CAM.04 (Δ*ahpCD*) (Fig. 6C). Probably due to high H_2_O_2_ levels, catalase activity in CAM.04 strain is induced as a compensatory effect. This concerted regulation between the two H_2_O_2_ scavenger enzymes has been already reported for *Xanthomonas campestris* pv *phaseoli* and *Staphylococcus aureus* [46], [47]. Although the activation of the H_2_O_2_-inducible catalase is clearly observed, the intracellular H_2_O_2_ levels in the mutant strain were still higher than in the wild-type. Simultaneously, a decrease in SOD activity is observed when compared to the wild-type. This can either be due to a compensatory effect towards re-establishing the cell redox status or to a SOD inactivation by H_2_O_2_ [39]. Regardless of the oxidative stress response triggered by the absence of AhpCD it should be noticed that CAM.04 presented a pimaricin overproducer phenotype that was reinforced by the addition of H_2_O_2_ to the culture broth during RG2 phase. A similar behaviour was observed in the CAM.05 (Δ*katA1*) strain. On the other hand, the high levels of intracellular H_2_O_2_ will trigger the production of the highly toxic hydroxyl radical as a consequence of the oxidation of unincorporated intracellular ferrous iron (Fenton reaction). The effect that the hydroxyl radicals may have on the pimaricin biosynthesis was assessed in CAM.04 iron-supplemented cultures. Although a negative effect could be expected due to the toxic nature of the hydroxyl radicals, no decrease in pimaricin specific production was observed. Instead there was an increase in iron-supplemented cultures that could be explained by the optimization of iron conditions in the growth medium.

Finally, the bald phenotype exhibited by the CAM.05 strain unveils a correlation between the H_2_O_2_-inducible catalase, KatA1, and morphological differentiation of *S. natalensis* on solid culture. Unlike liquid cultures where total catalase activity increases during the mid-late stationary growth phase, *S. natalensis* presents high catalase activity levels (from KatA1) during and after the emergence of aerial mycelia on solid culture (Beites T.B., unpublished results). It was suggested that during the development of aerial mycelia cells undergo a transient state of oxidative stress [48], [49] that could result from the nutrition limitation and other signals that trigger morphological differentiation or from a higher exposure to oxygen by aerial mycelia. The absence of the H_2_O_2_ inducible catalase in CAM.05 severely hampers the ability of the strain to cope with induced oxidative stress which may prevent a normal morphological differentiation of *S. natalensis* on solid cultures.

From an industrial point of view, oxygen supply is a key parameter on the production of secondary metabolites. While low O_2_ concentrations limit growth and product formation, increased O_2_ concentration usually can improve secondary metabolite production [50], [51]. However when above a certain threshold, O_2_ may have regulating and toxic effects on microbial cultures mainly due to induction of oxidative stress. Like other environment induced responses, the oxidative stress related mechanisms are embedded in global networks that extend the effect of the oxidative stress response into secondary metabolism. Thus, it is reasonable to assume that oxygen, or the by-products of its reduction, may have a regulatory effect on the *Streptomyces* secondary metabolism. The results obtained in this work point out to an important role of H_2_O_2_ intracellular levels towards the regulation of pimaricin biosynthesis, presumably through redox-based mechanisms. Maintaining H_2_O_2_ intracellular levels, particularly during late exponential – early stationary phases, proved to be important to elicit pimaricin biosynthesis. Although the punctual H_2_O_2_ modulation achieved by the addition of exogenous H_2_O_2_ suggested such a crosstalk, the correlation between H_2_O_2_ and secondary metabolism has been clearly shown by the characterization of the H_2_O_2_-related enzymes defective mutants, in which a long-term modulation of H_2_O_2_ levels is achieved.

## Materials and Methods

### Bacterial strains, plasmids and growth conditions

The strains, plasmids, and cosmids used in this study are listed in Table 1. *S. natalensis* was routinely grown by inoculating a spore suspension (3×10^8^ c.f.u.) into 100 mL of YEME medium [52] without sucrose and prepared with deionized water, in 1 L baffled flasks. Cultures were incubated in an orbital incubator shaker at 200 r.p.m. and 30 °C for 72 hours. Sporulation was achieved in TBO medium [10]. Induced oxidative stress studies were carried out by challenging cultures with 1 mM H_2_O_2_ over the different growth phases which were defined by the change in the rate of increase in cell density at 600 nm [21], [22]. Growth phases were defined as follows: early exponential (RG1) between OD_600nm_ 1.5 and 4; late exponential (RG2) between OD_600nm_ 5 and 8; stationary phase with pimaricin production (S/P) between 38 h and 48 h of culture and late exponential without pimaricin production (S/NP) at 72 h after inoculation. *E. coli* cells were routinely grown in LB medium.

### Nucleic acids and protein procedures

Standard genetic techniques with *Streptomyces, E. coli* and *in vitro* DNA manipulations were carried out as previously described [52], [53]. Isolation of total DNA from *Streptomyces* was performed using the MasterPure^TM^ Gram Positive DNA Purification Kit (Epicentre). Southern hybridization was carried out with probes labelled with digoxigenin by using the DIG DNA labelling kit (Roche).

For *S. natalensis* total RNA isolation 1 ml-aliquots of cultures grown in YEME medium were mixed with two volumes of RNA Protect Bacteria Reagent (Qiagen). Mycelia were harvested by centrifugation and immediately frozen by immersion in liquid nitrogen. Buffer RLT (Qiagen) in the presence of 1.0% (v/v) β-mercaptoethanol was added to the frozen mycelium, mixed thoroughly and cells broken by sonication (Branson Sonifier, Model B-15). RNeasy Mini Spin columns were used for RNA isolation according to manufacturer's instructions. DNA was removed by two serial DNase treatments, an in-column DNase I RNase-free (Qiagen), followed by a batch treatment using the DNA-free Kit (Ambion). Total RNA concentration was determined with a NanoDrop ND-1000 spectrophotometer (Thermo Scientific), and RNA quality and integrity were checked in an Experion^TM^ Automated Electrophoresis System (Bio-Rad).

For *S. natalensis* crude protein extracts preparation, mycelia from 1 mL of culture were harvested by centrifugation. Cell pellets were washed once with 50 mM K-phosphate buffer, pH 6.8, and resuspended in 0.5 mL of the same buffer containing 25% (v/v) of a protease inhibitor (Roche). Cells were disrupted by sonication (Branson Sonifier, Model B-15), the lysate was centrifuged and the pellet discarded. Protein content of cellular extracts was determined by the BCA^TM^ Protein Assay Kit (Pierce) using bovine serum albumin as a standard.

Intergeneric conjugation between *E. coli* ET12567 [pUZ8002] and *S. natalensis* ATCC 27448 was performed as previously described [35].

### Determination of catalase and superoxide dismutase activities

Enzymatic assays were performed to determine total catalase and superoxide dismutase (SOD) activity in *S. natalensis* protein extracts. Catalase activity was quantified spectrophotometrically by following the rate of decrease in absorbance at 240 nm caused by the disappearance of H_2_O_2_ [54]. The assay mix contained 30 µL of protein extract and 10 mM H_2_O_2_ in 50 mM phosphate buffer pH 6.8 in a final volume of 1 mL. Assays were carried out at 25 °C. One unit of enzyme activity is defined as the amount required for the conversion of 1 µmol substrate into product per min. SOD activity was assayed by the method previously described [39], [55]. One unit of enzyme activity was defined as the amount of SOD required to inhibit the reduction of nitroblue tetrazolium by 50% under the reaction conditions.

Catalase, peroxidase and superoxide dismutase activities of *S. natalensis* protein extracts were also analyzed on 7.5% –10% (w/v) non-denaturing polyacrylamide gels. Catalase activity was visualized on the gel using the method previously described [41], [56]. Peroxidase gel activity was detected using a mixture of chromogenic substrates [57]. SOD activity was detected on native gels by its ability to deplete superoxide, which can reduce nitroblue tetrazolium [39], [58].

### Two-dimensional (2D) gel electrophoresis (PAGE)

2D electrophoresis was performed as previously described [59]. A total of 150 µg of crude protein extract was treated with 3% (v/v) of Benzonase Nuclease (Sigma) at 37 °C for 30 min, subjected to a clean-up process using the 2-D Clean up Kit (GE Healthcare), and used for IEF in 17-cm precast IPG strips (Bio-Rad) with linear pH gradient of 4.0–7.0 using a PROTEAN IEF Cell (Bio-Rad). Second dimension was run in 12.5% (w/v) SDS-PAGE gels using an Ettan Dalt apparatus (GE Healthcare) as recommended by the manufacturer. Gels were silver stained following an MS-compatible protocol [60]. LMW low molecular weight marker (GE Healthcare) was used as size markers. Image analysis was performed with biological triplicates by using the PDQuest 2-D analysis software (Bio-Rad).

Protein spots were excised from gels and digested with trypsin. Samples were analyzed using the 4700 Proteomics Analyzer MALDI-TOF/TOF (Applied Biosystems) as described previously (Pinho *et al.*, 2009). Data were analyzed using GPS Explorer (Version 3.6; Applied Biosystems). The alkyl hydroperoxide reductase C, ahpC, protein spot was identified using combined data from PMF (Peptide Mass Fingerprint) and tandem mass (MS/MS) spectra. The Mascot (Matrix Science, UK) protein identification algorithm, which reflects these two levels of information, had a score associated with this identification of 155 with an expected value of 2.9e-009, and a confidence interval of 100%. Two tryptic peptide peaks have been selected for MS/MS peptide sequencing. The sequences of the fragmented peptides in the identified protein were LNDEFADR and ALGIEGEDGFAQR.

### Quantification of intracellular reactive oxygen species (ROS)

Levels of intracellular H_2_O_2_ were detected with dihydrorhodamine 123 (DHR) (Invitrogen). Aliquots from the culture broth were taken at selected time points, pellets were resuspended in 50 mM K-phosphate buffer pH 6.8 and DHR was added to a final concentration of 15 µg ml^−1^. Cells were incubated for 60 min at 30 °C in the dark. Mycelia were washed twice in 50 mM K-phosphate buffer pH 6.7 and cells were broken by sonication. The amount of ROS was quantified with a spectrofluorometer (Fluoromax-4, Horiba) emitting at 504 nm and measuring at 534 nm. Protein content of the crude extracts was used as normalization factor.

### PCR and RT-qPCR

DNA amplification by PCR was performed with GoTaq Flexi DNA Polymerase (Promega) or Pfu DNA polymerase (Fermentas) according to the manufacturer's instructions. For gene expression studies 1 µg of DNase I-treated (DNA-free Kit, Ambion) total RNA was transcribed with the iScript™ Select cDNA Synthesis Kit (Bio-Rad), using the random primers supplied, and following the manufacturer's instructions. qPCR amplifications were performed using the primer pairs listed in Table S1 and using 0.2 µM of each primer,10 µl of iQ™ SYBR® Green Supermix (Bio-Rad) and 2 µl of template cDNA. qPCRs were carried out in the iCycler iQ5 Real-Time PCR detection system (Bio-Rad) and conditions were as follows: 95°C for 5 min, 40 cycles of 95°C for 30 sec, 55, 60 or 65 °C (depending of the set of primers used) for 30 sec, and 72 °C for 30 sec. Standard dilutions of the cDNA were used to check the relative efficiency and quality of primers. Negative controls (no template cDNA) were included in all qPCR. A melting curve analysis was performed at the end of each qPCR assay to exclude the formation of nonspecific products. The data obtained were analyzed using the method described in Pfaffl [61]. For each analysis 16 S rRNA and *lysA* were used for normalization. The identity of each amplified product was corroborated by sequencing the PCR product.

### Gene identification, cloning and sequencing

For the identification of the antioxidant enconding genes (monofunctional catalase, SOD and the alkyl hydroperoxidase system) of *S. natalensis*, internal hybridization probes were obtained by PCR amplification using genomic DNA of *S. natalensis* as template. Primer pairs were designed based on alignments of known nucleotide sequences of these proteins from *S. avermitilis*, *S. coelicolor* and *S. griseus*. Once confirmed the genetic identity of the PCR products by sequencing, they were labelled with digoxigenin and used as probes for screening a *S. natalensis* ATCC 27448 cosmid library [10].

Plasmid DNA was isolated from *E. coli* cultures using the GenElute™ Plasmid Miniprep Kit (Sigma-Aldrich, Saint Louis, MO), and sequenced at STAB Vida (Lisbon). Each nucleotide was sequenced a minimum of three times independently on both strands. Alignment of sequence contigs was performed using the Vector NTI ContigExpress program (Invitrogen). DNA and protein sequences were analyzed with the NCBI World Wide Web BLAST server.

Published sequences were retrieved from GenBank and computer-assisted sequence comparisons were performed using Vector NTI Advance 10 (Invitrogen).

### Construction of mutant strains and complementation

The strategy used for gene disruption was based on the PCR targeting system developed in *S. coelicolor* [34]. The coding sequences of *sodF*, *ahpCD* and *katA1* genes from *S. natalensis* ATCC 27448 were replaced by the *aac(3)IV/oriT* cassette from plasmid pIJ773. The primers used for amplifying the cassette are listed in Table 2. The PCR targeting strategy originated mutant stains CAM.02, CAM.04 and CAM.05 respectively. The identity of all mutants was confirmed by Southern hybridization and PCR. Complementation of defective mutants was carried out by inserting into the integrative vector pSET152neo a DNA fragment containing the wild-type gene (either *ahpCD* or *sodF*) plus their own promoters. The originating plasmids (pSET*ahpCD* and pSET*sodF*) were transfered to *S. natalensis* CAM.04 and CAM.02 respectively, by conjugation. pSET152neo was also introduced into *S. natalensis* wild-type as control.

### Pimaricin Production

The production of pimaricin in liquid cultures was routinely quantified by spectrophotometric determination at 304 nm. A 100 µL aliquot of culture was extracted with 8 vol of butanol, and the organic phase was diluted in water-saturated butanol to bring the absorbance at 304 nm in the range of 0.1–0.8 U. Pimaricin was quantified using a solution of pure pimaricin (Calbiochem) as standard. To confirm the identity of pimaricin, a UV–visible absorption spectrum (absorption peaks at 319, 304, 291 and 281 nm) was routinely determined. The fungicidal activity of pimaricin was tested by bioassay using *C. utilis* CECT 1061 as test organism. In addition to spectrophotometric determination, mutants broth extracts were also analysed by UPLC using a Waters ACQUITY System coupled to a PDA detector, fitted with a reverse-phase BEH C18 column (2.1×50 mm, particle size, 1.7 µm.). Elution was performed with a methanol/water gradient (0.4 ml/min) according to the following program (methanol concentration): 50% 0–0.73 min, up to 90% 0.73–2.90 min, 90% 2.90–4.83, down to 50% 4.83–6.02 min and 50% 6.02–7.21 min. Under these conditions pimaricin eluted at 2.60 min.

### Nucleotide sequence accession numbers

The sequence data associated with this study have been submitted to the GenBank database under accession numbers JN005772, JN005773 and JN005774.

## Supporting Information

Figure S1 **Total catalase activity of** ***S. natalensis*** **ATCC 27448,** ***S. coelicolor*** **M145 and** ***S. avermitilis*** **in YEME medium.** Samples were collected at the four defined growth phases (see Experimental Procedures): RG1, RG2, S/P and S/NP. Results (average of triplicates and standard deviation) are representative of three independent experiments.
(TIF)

Figure S2 **Construction of strain CAM.05 by** **gene replacement of** ***katA1*** **.** A) Predicted restriction enzyme polymorphism caused by gene replacement. The NcoI-XbaI restriction pattern before and after replacement is shown. The probe used for southern hybridization is indicated by thick lines. N, NcoI; X, XbaI. B) Confirmation of gene disruption by PCR. A pair of primers, cKatA_F and ckatA_R, covering the deleted region in the chromosome were used for quick screening to identify double crossover mutants. C) Confirmation of gene disruption by Southern hybridization of the NcoI-XbaI digested chromosomal DNA of the wild type (lane 1), and CAM.05 (ΔkatA1; lane 2) strains. Lane M, DIG-labeled DNA Molecular Weight Marker II (Roche). Extra bands hybridizing on the Southern blot are cross-hybridization with other genomic fragments.
(TIF)

Figure S3 **Construction of strain CAM.02 by gene replacement of** ***sodF*** **.** A) Predicted restriction enzyme polymorphism caused by gene replacement. The NotI restriction pattern before and after replacement is shown. The probe used for southern hybridization is indicated by thick line. A, Apa I; N, NotI. B) Confirmation of gene disruption by PCR. A pair of primers, CsodF-F and CsodF-R, covering the deleted region in the chromosome were used for quick screening to identify double crossover mutants. C) Confirmation of gene disruption by Southern hybridization of the NotI digested chromosomal DNA of the wild type (lane 1), and CAM.02 (Δ*sodF*; lane 2) strains. Lane L, DIG-labeled DNA Molecular Weight Marker II (Roche).
(TIF)

Figure S4 **Construction of strain CAM.04 by gene replacement of** ***ahpCD*** **.** A) Predicted restriction enzyme polymorphism caused by gene replacement. The PvuII restriction pattern before and after replacement is shown. The probe used for southern hybridization is indicated by thick lines. B, BamHI; N, NcoI; P, PvuII. B) Confirmation of gene disruption by PCR. A pair of primers, Cahp-F and Cahp-R, covering the deleted region in the chromosome were used for quick screening to identify double crossover mutants. C) Confirmation of gene disruption by Southern hybridization of the PvuII digested chromosomal DNA of the wild type (lane 1), and CAM.04 (Δ*ahpCD*; lane 2) strains. Lane L, DIG-labeled DNA Molecular Weight Marker II (Roche).
(TIF)

Table S1 **Primers used in this work.**
(PDF)

## References

[1] Berdy J (2005). Bioactive microbial metabolites.. J Antibiot (Tokyo).

[2] Bibb M (2005). Regulation of secondary metabolism in streptomycetes.. Current Opinion in Microbiology 8:.

[3] Storz G, Imlay JA (1999). Oxidative stress.. Curr Opin Microbiol.

[4] Rhee SG (2006). Cell signaling. H_2_O_2_, a necessary evil for cell signaling.. Science.

[5] Zheng M, Aslund F, Storz G (1998). Activation of the OxyR transcription factor by reversible disulfide bond formation.. Science.

[6] Hidalgo E, Ding H, Demple B (1997). Redox signal transduction: mutations shifting [2Fe-2S] centers of the SoxR sensor-regulator to the oxidized form.. Cell.

[7] Hahn JS, Oh SY, Chater KF, Cho YH, Roe JH (2000). H2O2-sensitive fur-like repressor CatR regulating the major catalase gene in *Streptomyces coelicolor*.. J Biol Chem.

[8] Kwon HJ, Kim SU (1999). Molecular basis for enhanced biosynthesis of clavulanic acid by a redox-cycling agent, phenazine methosulfate, in *Streptomyces clavuligerus*.. Appl Microbiol Biotechnol.

[9] Kwon HJ, Kim SU (1998). Enhanced biosynthesis of clavulanic acid in *Streptomyces clavuligerus* due to oxidative challenge by redox-cycling agents.. Appl Microbiol Biotechnol.

[10] Aparicio JF, Colina AJ, Ceballos E, Martin JF (1999). The biosynthetic gene cluster for the 26-membered ring polyene macrolide pimaricin. A new polyketide synthase organization encoded by two subclusters separated by functionalization genes.. J Biol Chem.

[11] te Welscher YM, Jones L, van Leeuwen MR, Dijksterhuis J, de Kruijff B (2010). Natamycin inhibits vacuole fusion at the priming phase via a specific interaction with ergosterol.. Antimicrob Agents Chemother.

[12] Martin JF, Aparicio JF (2009). Enzymology of the polyenes pimaricin and candicidin biosynthesis.. Methods Enzymol.

[13] Anton N, Mendes MV, Martin JF, Aparicio JF (2004). Identification of PimR as a positive regulator of pimaricin biosynthesis in *Streptomyces natalensis*.. J Bacteriol.

[14] Anton N, Santos-Aberturas J, Mendes MV, Guerra SM, Martin JF (2007). PimM, a PAS domain positive regulator of pimaricin biosynthesis in *Streptomyces natalensis*.. Microbiology.

[15] Mendes MV, Recio E, Anton N, Guerra SM, Santos-Aberturas J (2007). Cholesterol oxidases act as signaling proteins for the biosynthesis of the polyene macrolide pimaricin.. Chem Biol.

[16] Mendes MV, Recio E, Fouces R, Luiten R, Martin JF (2001). Engineered biosynthesis of novel polyenes: a pimaricin derivative produced by targeted gene disruption in *Streptomyces natalensis*.. Chem Biol.

[17] Vicente CM, Santos-Aberturas J, Guerra SM, Payero TD, Martin JF (2009). PimT, an amino acid exporter controls polyene production via secretion of the quorum sensing pimaricin-inducer PI-factor in *Streptomyces natalensis*.. Microb Cell Fact.

[18] Aparicio JF, Fouces R, Mendes MV, Olivera N, Martin JF (2000). A complex multienzyme system encoded by five polyketide synthase genes is involved in the biosynthesis of the 26-membered polyene macrolide pimaricin in *Streptomyces natalensis*.. Chem Biol.

[19] Santos-Aberturas J, Vicente CM, Guerra SM, Payero TD, Martin JF (2011). Molecular control of polyene macrolide biosynthesis: direct binding of the regulator PimM to eight promoters of pimaricin genes and identification of binding boxes.. The Journal of biological chemistry.

[20] Mendes MV, Tunca S, Anton N, Recio E, Sola-Landa A (2007). The two-component phoR-phoP system of *Streptomyces natalensis*: Inactivation or deletion of phoP reduces the negative phosphate regulation of pimaricin biosynthesis.. Metab Eng.

[21] Puglia AM, Vohradsky J, Thompson CJ (1995). Developmental control of the heat-shock stress regulon in *Streptomyces coelicolor*.. Mol Microbiol.

[22] Huang J, Lih CJ, Pan KH, Cohen SN (2001). Global analysis of growth phase responsive gene expression and regulation of antibiotic biosynthetic pathways in *Streptomyces coelicolor* using DNA microarrays.. Genes Dev.

[23] Nieselt K, Battke F, Herbig A, Bruheim P, Wentzel A (2010). The dynamic architecture of the metabolic switch in *Streptomyces coelicolor*.. BMC Genomics.

[24] Cho YH, Roe JH (1997). Isolation and expression of the catA gene encoding the major vegetative catalase in *Streptomyces coelicolor* Muller.. J Bacteriol.

[25] Walker GE, Dunbar B, Hunter IS, Nimmo HG, Coggins JR (1995). A catalase from *Streptomyces coelicolor* A3(2).. Microbiology.

[26] Zou P, Borovok I, Ortiz de Orue Lucana D, Muller D, Schrempf H (1999). The mycelium-associated *Streptomyces reticuli* catalase-peroxidase, its gene and regulation by FurS.. Microbiology.

[27] Seaver LC, Imlay JA (2001). Alkyl Hydroperoxide Reductase Is the Primary Scavenger of Endogenous Hydrogen Peroxide in *Escherichia coli*.. J Bacteriol.

[28] Lee JW, Helmann JD (2006). The PerR transcription factor senses H_2_O_2_ by metal-catalysed histidine oxidation.. Nature.

[29] Lee JW, Helmann JD (2006). Biochemical characterization of the structural Zn^2+^ site in the *Bacillus subtilis* peroxide sensor PerR.. J Biol Chem.

[30] Parker MW, Blake CC (1988). Iron- and manganese-containing superoxide dismutases can be distinguished by analysis of their primary structures.. FEBS Lett.

[31] Stallings WC, Pattridge KA, Strong RK, Ludwig ML (1985). The structure of manganese superoxide dismutase from *Thermus thermophilus* HB8 at 2.4-A resolution.. J Biol Chem.

[32] Kim JS, Jang JH, Lee JW, Kang SO, Kim KS (2000). Identification of cis site involved in nickel-responsive transcriptional repression of sodF gene coding for Fe- and Zn-containing superoxide dismutase of *Streptomyces griseus*.. Biochim Biophys Acta.

[33] Hillas PJ, del Alba FS, Oyarzabal J, Wilks A, Ortiz De Montellano PR (2000). The AhpC and AhpD antioxidant defense system of *Mycobacterium tuberculosis*.. J Biol Chem.

[34] Gust B, Challis GL, Fowler K, Kieser T, Chater KF (2003). PCR-targeted *Streptomyces* gene replacement identifies a protein domain needed for biosynthesis of the sesquiterpene soil odor geosmin.. Proc Natl Acad Sci U S A.

[35] Enriquez LL, Mendes MV, Anton N, Tunca S, Guerra SM (2006). An efficient gene transfer system for the pimaricin producer *Streptomyces natalensis*.. FEMS Microbiol Lett.

[36] el-Enshasy HA, Farid MA, el-Sayed el SA (2000). Influence of inoculum type and cultivation conditions on natamycin production by *Streptomyces natalensis*.. J Basic Microbiol.

[37] Cho YH, Lee EJ, Roe JH (2000). A developmentally regulated catalase required for proper differentiation and osmoprotection of *Streptomyces coelicolor*.. Mol Microbiol.

[38] Chung HJ, Kim EJ, Suh B, Choi JH, Roe JH (1999). Duplicate genes for Fe-containing superoxide dismutase in *Streptomyces coelicolor* A3(2).. Gene.

[39] Kim FJ, Kim HP, Hah YC, Roe JH (1996). Differential expression of superoxide dismutases containing Ni and Fe/Zn in *Streptomyces coelicolor*.. Eur J Biochem.

[40] Kim EJ, Chung HJ, Suh B, Hah YC, Roe JH (1998). Expression and regulation of the sodF gene encoding iron- and zinc-containing superoxide dismutase in *Streptomyces coelicolor* Muller.. J Bacteriol.

[41] Kim H, Lee JS, Hah YC, Roe JH (1994). Characterization of the major catalase from *Streptomyces coelicolor* ATCC 10147.. Microbiology.

[42] Hahn JS, Oh SY, Roe JH (2002). Role of OxyR as a peroxide-sensing positive regulator in *Streptomyces coelicolor* A3(2).. J Bacteriol.

[43] Forman HJ, Maiorino M, Ursini F (2010). Signaling functions of reactive oxygen species.. Biochemistry.

[44] Taylor BL, Zhulin IB (1999). PAS domains: internal sensors of oxygen, redox potential, and light.. Microbiology and molecular biology reviews: MMBR.

[45] Kanth BK, Jnawali HN, Niraula NP, Sohng JK (2010). Superoxide dismutase (SOD) genes in *Streptomyces peucetius*: Effects of SODs on secondary metabolites production.. Microbiol Res.

[46] Mongkolsuk S, Whangsuk W, Vattanaviboon P, Loprasert S, Fuangthong M (2000). A Xanthomonas alkyl hydroperoxide reductase subunit C (ahpC) mutant showed an altered peroxide stress response and complex regulation of the compensatory response of peroxide detoxification enzymes.. J Bacteriol.

[47] Cosgrove K, Coutts G, Jonsson IM, Tarkowski A, Kokai-Kun JF (2007). Catalase (KatA) and alkyl hydroperoxide reductase (AhpC) have compensatory roles in peroxide stress resistance and are required for survival, persistence, and nasal colonization in *Staphylococcus aureus*.. J Bacteriol.

[48] Chater KF (2001). Regulation of sporulation in *Streptomyces coelicolor* A3(2): a checkpoint multiplex?. Current Opinion in Microbiology.

[49] Nystrom T (1999). Starvation, cessation of growth and bacterial aging.. Current Opinion in Microbiology.

[50] Song SK, Jeong YS, Kim PH, Chun GT (2006). Effects of dissolved oxygen level on avermectin B-1a production by *Streptomyces avermitilis* in computer-controlled bioreactor cultures.. Journal of Microbiology and Biotechnology.

[51] Yegneswaran PK, Gray MR, Thompson BG (1991). Effect of dissolved oxygen control on growth and antibiotic production in *Streptomyces clavuligerus* fermentations.. Biotechnol Prog.

[52] Kieser T, Bibb MJ, Buttner MJ, Chater KF, Hopwood DA (2000). Practical *Streptomyces* Genetics..

[53] Sambrook J, Russell DW (2001). Molecular Cloning: a laboratory manual..

[54] Beers RF, Sizer IW (1952). A spectrophotometric method for measuring the breakdown of hydrogen peroxide by catalase.. J Biol Chem.

[55] Beauchamp C, Fridovich I (1971). Superoxide dismutase: improved assays and an assay applicable to acrylamide gels.. Anal Biochem.

[56] Clare DA, Duong MN, Darr D, Archibald F, Fridovich I (1984). Effects of molecular oxygen on detection of superoxide radical with nitroblue tetrazolium and on activity stains for catalase.. Anal Biochem.

[57] Conyers SM, Kidwell DA (1991). Chromogenic substrates for horseradish peroxidase.. Anal Biochem.

[58] Steinman HM (1985). Bacteriocuprein superoxide dismutases in pseudomonads.. J Bacteriol.

[59] Gorg A, Obermaier C, Boguth G, Harder A, Scheibe B (2000). The current state of two-dimensional electrophoresis with immobilized pH gradients.. Electrophoresis.

[60] Gromova I, Celis JE, Celis JE, Carter N, Simons K, Small J, Hunter T (2006). Protein Detection in Gels by Silver Staining: A Procedure Compatible with Mass Spectrometry.. Cell Biology: A Laboratory Handbook. 3rd ed.

[61] Pfaffl MW (2001). A new mathematical model for relative quantification in real-time RT-PCR.. Nucleic Acids Res.

[62] Struyk AP, Hoette I, Drost G, Waisvisz JM, van Eek T (1958). Pimaricin, a new antifungal antibiotic.. Antibiotics Annual.

[63] Hanahan D (1983). Studies on transformation of *Escherichia coli* with plasmids.. J Mol Biol.

[64] MacNeil DJ, Gewain KM, Ruby CL, Dezeny G, Gibbons PH (1992). Analysis of *Streptomyces avermitilis* genes required for avermectin biosynthesis utilizing a novel integration vector.. Gene.

[65] Datsenko KA, Wanner BL (2000). One-step inactivation of chromosomal genes in *Escherichia coli* K-12 using PCR products.. Proc Natl Acad Sci U S A.

[66] Cherepanov PP, Wackernagel W (1995). Gene disruption in *Escherichia coli*: TcR and KmR cassettes with the option of Flp-catalyzed excision of the antibiotic-resistance determinant.. Gene.

[67] Paget MS, Chamberlin L, Atrih A, Foster SJ, Buttner MJ (1999). Evidence that the extracytoplasmic function sigma factor sigmaE is required for normal cell wall structure in Streptomyces coelicolor A3(2).. J Bacteriol.

