# Supplementary figures and images for: Crosstalk between ROS Homeostasis and Secondary Metabolism in S. natalensis ATCC 27448: Modulation of Pimaricin Production by Intracellular ROS
Source: PLoS One. 2011 Nov 17;6(11):e27472. doi: 10.1371/journal.pone.0027472 (PMC3219662; doi:10.1371/journal.pone.0027472)

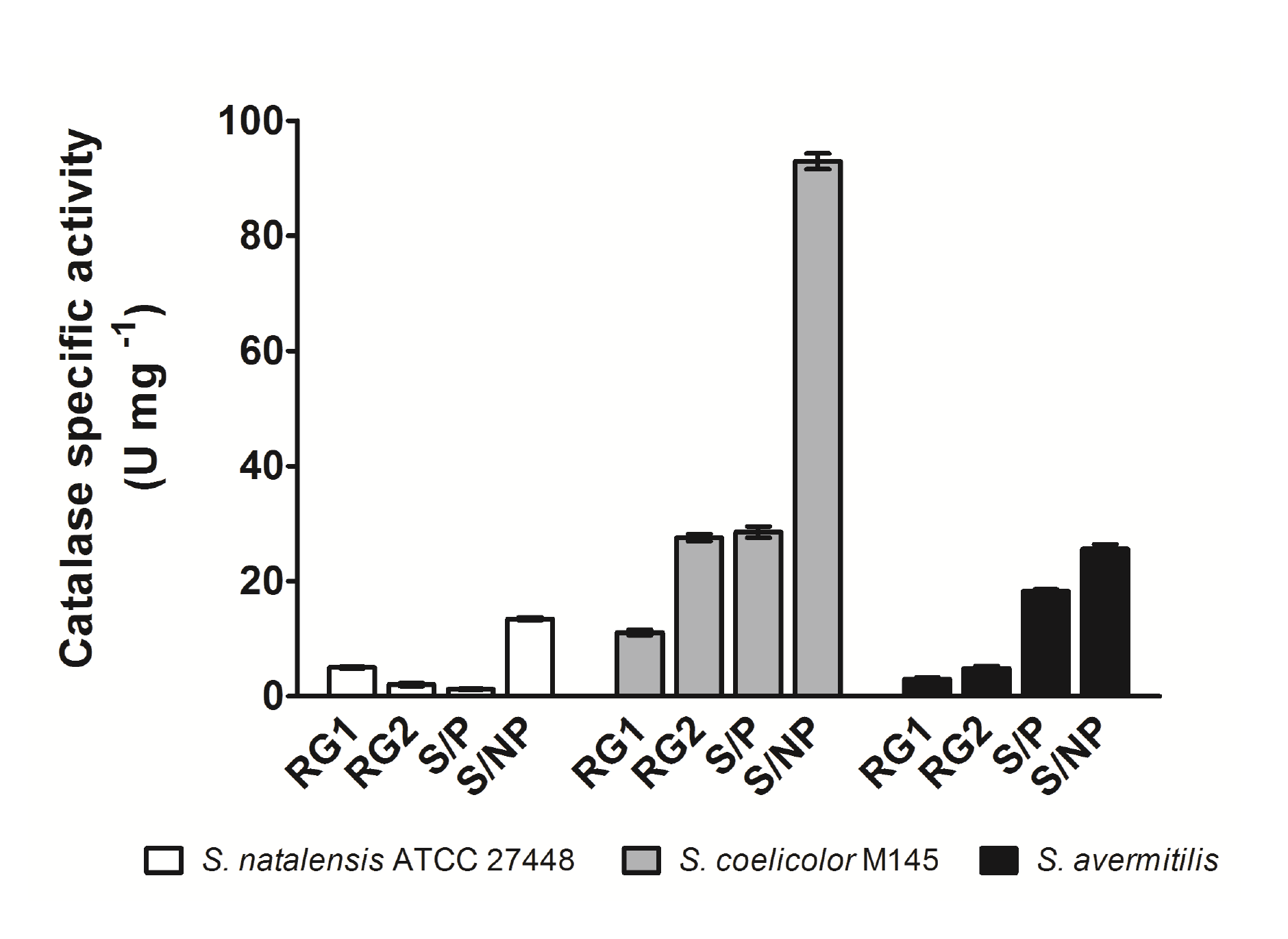

Supplement: Figure S1 — Total catalase activity of S. natalensis ATCC 27448, S. coelicolor M145 and S. avermitilis in YEME medium. Samples were collected at the four defined growth phases (see Experimental Procedures): RG1, RG2, S/P and S/NP. Results (average of triplicates and standard deviation) are representative of three independent experiments. (TIF) [file pone.0027472.s001.tif]

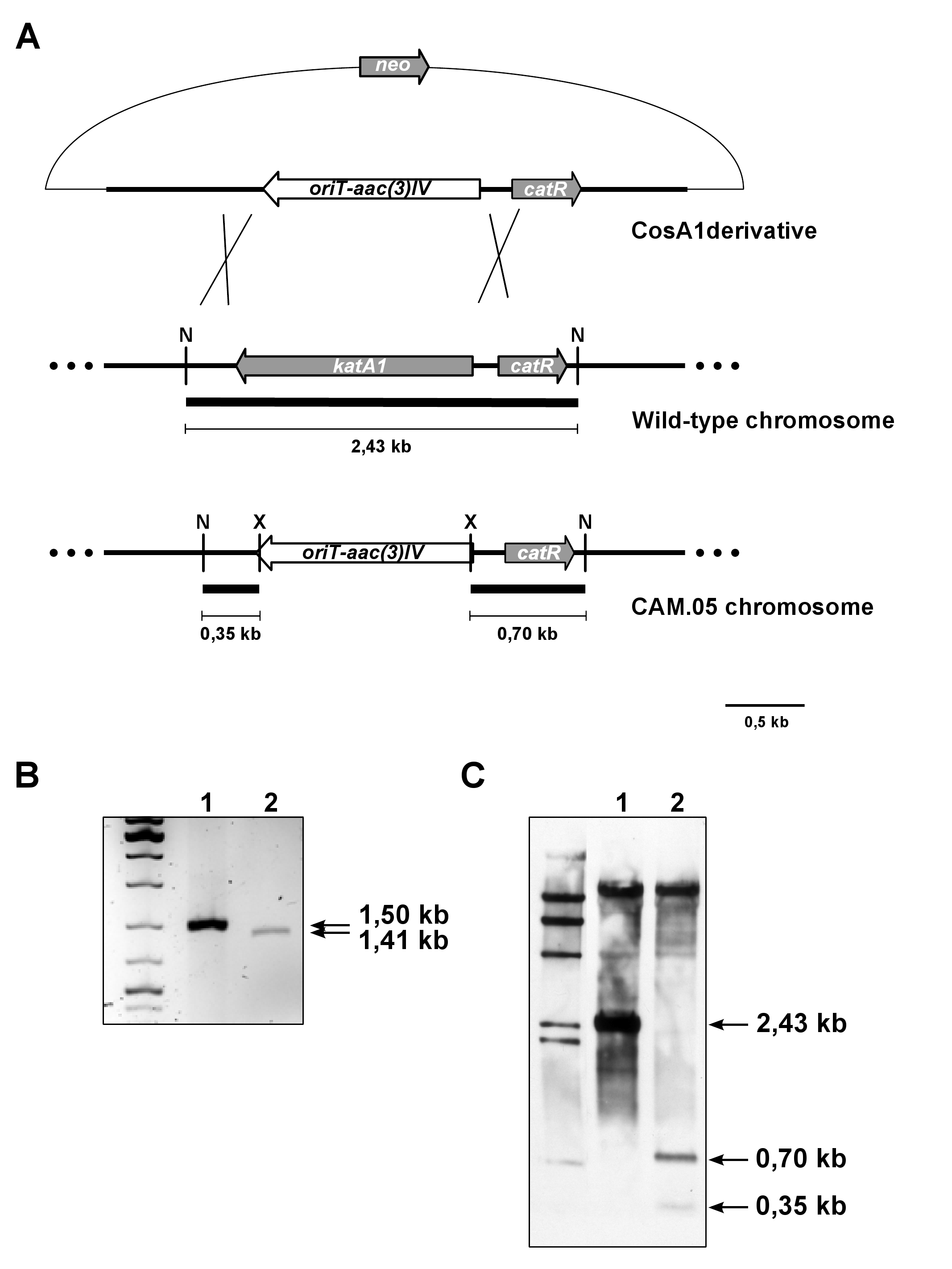

Supplement: Figure S2 — Construction of strain CAM.05 by gene replacement of katA1 . A) Predicted restriction enzyme polymorphism caused by gene replacement. The NcoI-XbaI restriction pattern before and after replacement is shown. The probe used for southern hybridization is indicated by thick lines. N, NcoI; X, XbaI. B) Confirmation of gene disruption by PCR. A pair of primers, cKatA_F and ckatA_R, covering the deleted region in the chromosome were used for quick screening to identify double crossover mutants. C) Confirmation of gene disruption by Southern hybridization of the NcoI-XbaI digested chromosomal DNA of the wild type (lane 1), and CAM.05 (ΔkatA1; lane 2) strains. Lane M, DIG-labeled DNA Molecular Weight Marker II (Roche). Extra bands hybridizing on the Southern blot are cross-hybridization with other genomic fragments. (TIF) [file pone.0027472.s002.tif]

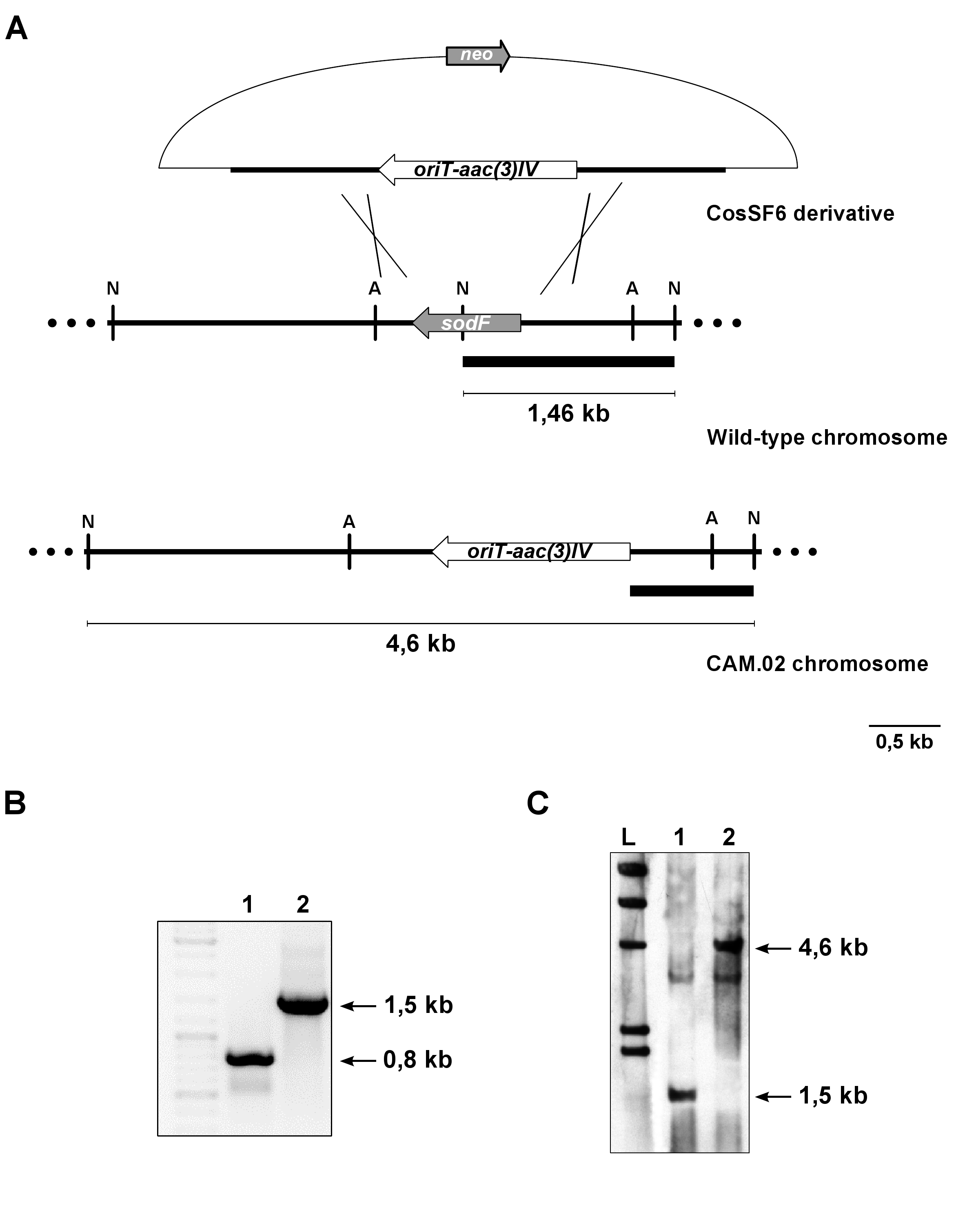

Supplement: Figure S3 — Construction of strain CAM.02 by gene replacement of sodF . A) Predicted restriction enzyme polymorphism caused by gene replacement. The NotI restriction pattern before and after replacement is shown. The probe used for southern hybridization is indicated by thick line. A, Apa I; N, NotI. B) Confirmation of gene disruption by PCR. A pair of primers, CsodF-F and CsodF-R, covering the deleted region in the chromosome were used for quick screening to identify double crossover mutants. C) Confirmation of gene disruption by Southern hybridization of the NotI digested chromosomal DNA of the wild type (lane 1), and CAM.02 (ΔsodF; lane 2) strains. Lane L, DIG-labeled DNA Molecular Weight Marker II (Roche). (TIF) [file pone.0027472.s003.tif]

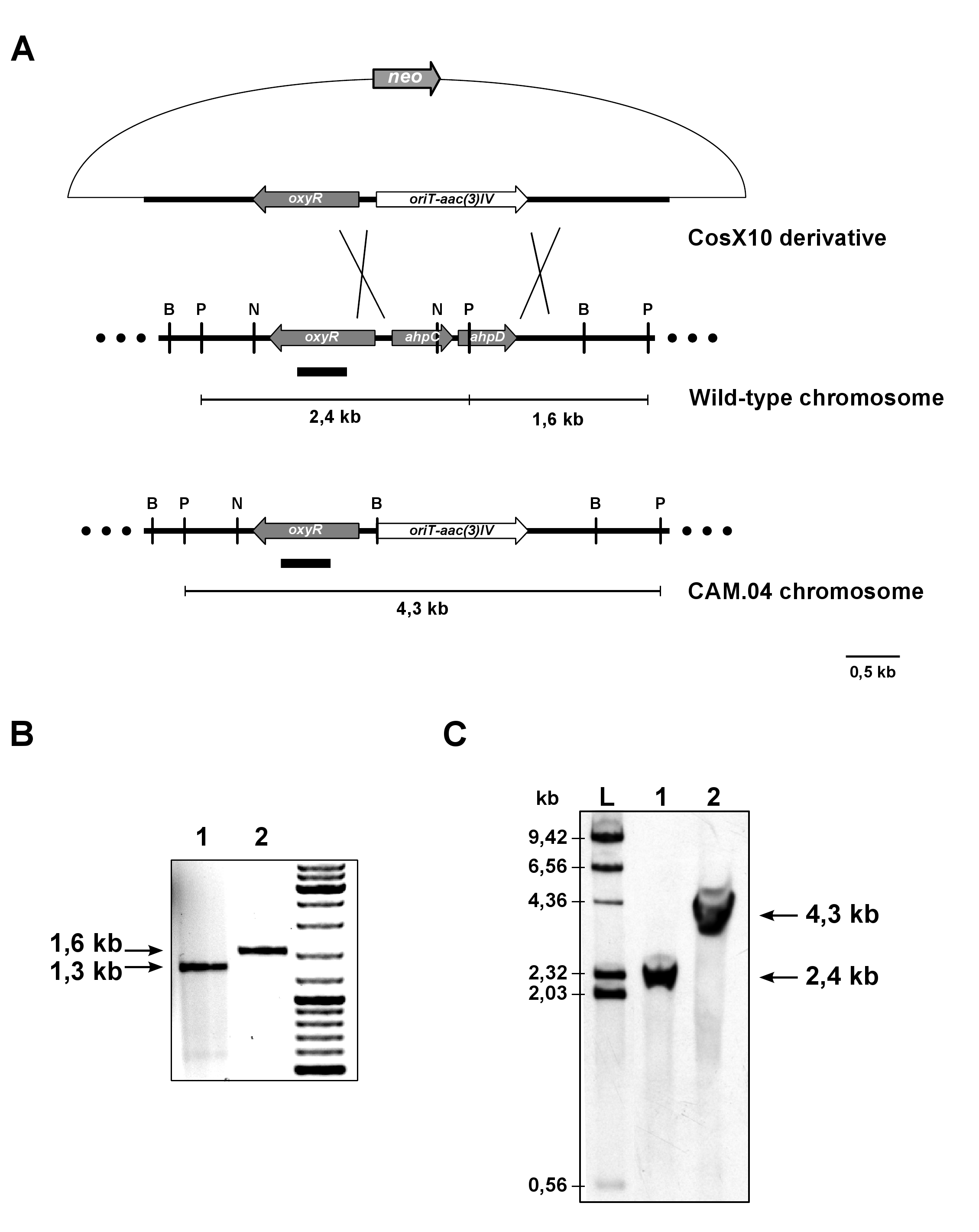

Supplement: Figure S4 — Construction of strain CAM.04 by gene replacement of ahpCD . A) Predicted restriction enzyme polymorphism caused by gene replacement. The PvuII restriction pattern before and after replacement is shown. The probe used for southern hybridization is indicated by thick lines. B, BamHI; N, NcoI; P, PvuII. B) Confirmation of gene disruption by PCR. A pair of primers, Cahp-F and Cahp-R, covering the deleted region in the chromosome were used for quick screening to identify double crossover mutants. C) Confirmation of gene disruption by Southern hybridization of the PvuII digested chromosomal DNA of the wild type (lane 1), and CAM.04 (ΔahpCD; lane 2) strains. Lane L, DIG-labeled DNA Molecular Weight Marker II (Roche). (TIF) [file pone.0027472.s004.tif]
