# Supplementary material for: Crosstalk between ROS Homeostasis and Secondary Metabolism in S. natalensis ATCC 27448: Modulation of Pimaricin Production by Intracellular ROS
Source: PLoS One. 2011 Nov 17;6(11):e27472. doi: 10.1371/journal.pone.0027472 (PMC3219662; doi:10.1371/journal.pone.0027472)
Supplement: Table S1 — Primers used in this work. (PDF) [file pone.0027472.s005.pdf]

Table S1. Oligonucleotides used in this study

Use (s)	Primer	Nucleotide Sequence (5' – 3')
**RT-qPCR**	ahpC-S	CTGGAGAAGGGCGAGGAG
	ahpC-AS	GGCACACGAAGGTGAAGTC
	oxyR-S	CGTCCCGAACATCACCGA
	oxyR-AS	CCGAGCCAGTGGTCCTTG
	katA1-S	CCTGGTCGGTCTTGAAGTGGTA
	katA1-AS	CTACGGCTCGCACACCTTC
	catR-S	CAACGCCCACCACGACCA
	catR-AS	TCGCCCTGGAGATGGACG
	16S-S	CAGGCTAGAGTTCGGTAGG
	16S-AS	CTCCTCAGCGTCAGTATCG
	lysA-S	AAACAGCACGACCAGCCC
	lysA-AS	CCGTGACCAGACGTCGTC
**Probes for genomic screening**	catAF	CGACCTSACCAAGGTSTGG
	catAR	TTGAYGCCGASGCGGTAG
	ahpC_F	CTGTCGGTGACAAGTTCCCC
	ahpC_R	GSTCCAGTTGCACGGGCA
	sodF_F	RYCCSSAGATCATCGAGC
	sodF_R	TGSAGRTAGAAGGCGTGC
**Gene deletion and confirmation**	TB1-F	CCAAGGGACAGGTGGAGATCACTTCAGGAGGCGCACG TGATTCCGGGGATCCGTCGACC
	TB1-R	CGAGAGCGCGCGGCGGTTTAGCCGCAAAGAGGCGTG CTATGTAGGCTGGAGCTGCTTC
	ccatA-F	GGTTTAGCCGCAAAGAGG
	ccatA-R	GATCACTTCAGGAGGCGC
	Mahp_F	CCCCCGCAGGTTTTTCTTTCGCTGCAAGGAGAGCGCGT GATTCCGGGGATCCGTCGACC
	Mahp_R	TCTTTGCGCAATGATCCGGCGCGCTGCCGACGGGAGT TATGTAGGCTGGAGCTGCTTC
	Cahp-F	CGTGTGGCACTGTTTGACCCG

	Cahp-R	ACCGCACCGGAACACGCA
	MsodF_F	ACTACTGCATTCACGCAGCTTGGAGGCGGGATCGGCATGATTCCGGGGATCCGTCGACC
	MsodF_R	AAGACGATCACGAGGCAGGACGATCAGAGGGCGCCGTCATGTAGGCTGGAGCTGCTTC
	CsodF-F	GCTCCGTCATTCCGCCTGC
	CsodF-R	GCGCCCGTCCGGTGATCA
**Complementation**	ahp-Fwp	CGGAATTCGCGCGACGGTGGGGATGACA
	ahp-Rvp	TCAAGCTTCCTCTAGACCGAACGACCCGAAACCGCA
	sodF-Fwp	CGGAATTCAGGCGCCGGAGAAGCTCGTG
	sodF-Rvp	TCAAGCTTCCTCTAGAATGTGCGGCTGCCAGGTGGGA
